# Lockdown surgery: the impact of coronavirus disease 2019 measures on cardiac cases

**DOI:** 10.1093/icvts/ivac060

**Published:** 2022-04-13

**Authors:** Felix Nägele, Clemens Engler, Michael Graber, Nina Remmel, Jakob Hirsch, Leo Pölzl, Rosalie Huber, Victor Schweiger, Juliane Kilo, Nikolaos Bonaros, Ivan Tancevski, Michael Grimm, Can Gollmann-Tepeköylü, Johannes Holfeld

**Affiliations:** 1 Department of Cardiac Surgery, Medical University of Innsbruck, Austria; 2 Department of Internal Medicine I, Medical University of Innsbruck, Austria; 3 Department of Internal Medicine II, Medical University of Innsbruck, Austria

**Keywords:** COVID-19, SARS-CoV-2, Lockdown, Cardiac Surgery

## Abstract

**OBJECTIVES:**

The need to ration medical equipment and interventions during the coronavirus disease 2019 pandemic translated to an ever-lengthening wait list for surgical care. Retrospective analysis of lockdowns is of high importance to learn from the current situation for future pandemics. This monocentric study assessed the impact of lockdown periods on cardiac surgery cases and outcomes.

**METHODS:**

The single-centre cross-sectional descriptive observational study was conducted to investigate the first lockdown period and the following post-lockdown period in comparison to the same periods during the previous 3 years at the Department of Cardiac Surgery at the Medical University of Innsbruck. Data were prospectively collected and retrospectively analysed from the department-specific quality management system. The primary objective was to compare the number of open-heart procedures between the prelockdown and the lockdown period. The secondary objectives were to analyse the characteristics and the outcomes of open-heart procedures.

**RESULTS:**

There were no differences in patient demographics. A significant decrease of 29% in weekly surgical procedures was observed during the lockdown period. The surgical case-mix was unaffected: The numbers of aortic valve replacements, coronary artery bypass grafts, mitral valve repair or replacement procedures and others remained stable. The urgency of cases increased significantly, and the general health conditions of patients appeared to be worse. However, outcomes were unchanged.

**CONCLUSIONS:**

By implementing a rational patient selection process, the quality of open-heart procedures was maintained even though patients who underwent surgery during lockdown were sicker and more symptomatic.

**Figure:**
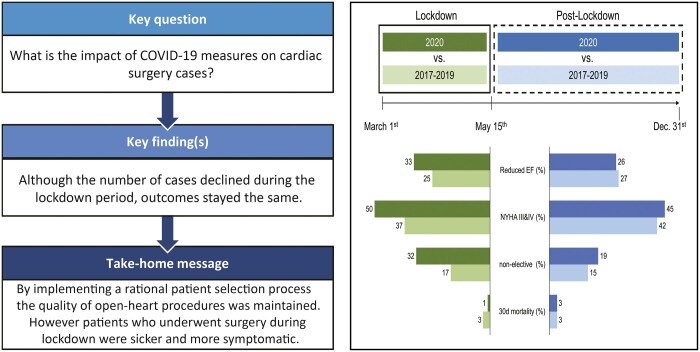

## INTRODUCTION

In late 2019 a new disease known as coronavirus disease 2019 (COVID-19), caused by severe acute respiratory syndrome coronavirus 2, emerged in Wuhan, China [1, 2]. Within 3 months, COVID-19 was announced as a global pandemic by the World Health Organization. Less than half a year after the first outbreak in Wuhan, more than 5 million people worldwide were infected, and more than 300,000 patients died [2, 3]. Overwhelmed by the large surges in COVID-19 cases, healthcare systems worldwide had to face limited space, supplies and ultimately insufficient intensive care unit (ICU) bed availability [1, 2, 4]. To contain the spread of the virus and ease the burden on hospitals, strict measures unprecedented in modern times were imposed. Entire nations have been placed under lockdown, schools and businesses closed and travel was restricted [5, 6]. In Austria, these measures against COVID-19 became obligatory on 16 March 2020. To prevent a collapse of medical resources, medical services in most medical specialties were required to be significantly reduced. Owing to the resource intensive nature of surgical care, multimillions of elective operations were canceled worldwide [7, 8]. After a heart operation, intensive care is a standard component of treatment for patients. Therefore, cardiac surgical programmes in Austria and other countries, e.g. Canada, were forced to prioritize urgent, emergency and salvage cases [3, 9, 10]. Beyond these limitations, an additional phenomenon of avoiding medical care emerged: From fear of getting infected, patients who would require cardiac surgery refrained from requesting help. This situation hypothetically leads to a drastic increase in cardiac surgery admissions, because those patients are more symptomatic and have more advanced cardiovascular diseases. The actual impact of this combination of an ever-lengthening wait list, limited resources and an increasing admission rate is yet to be understood [11]. Therefore, the goal of this study was to characterize the effect of COVID-19 on cardiac surgery by comparing the lockdown and post-lockdown periods with previous years at our institution. This retrospective analysis of early lockdowns is of high importance to learn from the current situation for future pandemics.

## PATIENTS AND METHODS

### Ethical statement

The study was conducted according to the guidelines of the Declaration of Helsinki and approved by the Ethics Committee of the Medical University of Innsbruck. The study was approved as a retrospective study with no need for written informed consent (Ethics Committee ID: 1265/2021 Version 4, Date: 20.09.2021).

## METHODS

We carried out a single-centre, cross-sectional descriptive observational study. Data were prospectively collected and retrospectively analysed from centralized hospital registries pertaining to the demographics, procedural volumes, urgency, type of surgery and outcome. Reports for all patients who required heart surgery on pump between 2017 and 2020 at the Medical University of Innsbruck were reviewed. No further exclusion criteria applied. A careful analysis of the patients’ baseline characteristics, operative data and postoperative outcomes was performed by 2 separate researchers. The data reviewed included hospitalization records and electronic quality control system entries. Throughout the study period, all procedures were performed and completed by the same surgical and anaesthesiology teams. The patients were divided into 2 groups based on the time at which they underwent the operation. First, the lockdown period group from the first lockdown period between 1 March and 15 May 2020 was compared to patients from the same period during the previous 3 years. Second, the post-lockdown period group from the period after the first lockdown, between 16 May and 31 December 2020, was compared to patients from the same period of the previous 3 years. The definition of these time periods was based on the implementation of non-pharmaceutical interventions at our department beginning 1 March and ending 15 May 2020. The societal lockdown period was chosen because, in many countries worldwide, the decision to announce a nationwide lockdown was based on COVID-19 occupied ICU capacities. The same was the case in Austria. The presence of unoccupied ICU beds is required to perform open-heart surgery because patients need intensive care following the procedures. Therefore, the nationwide decision on lockdown affected the cardiac surgery departments directly because they were introduced when ICU beds became scarce and ended when the situation improved.

The primary objective was to compare the number of open-heart procedures between the prelockdown and the lockdown periods. The secondary objectives were to analyse quantitative data on open-heart procedures to learn about the impact of healthcare resource shortages on cardiac surgery cases and of prioritization on outcome. Therefore, we selected a sound control cohort from the same months 3 years prior to the appearance of COVID-19. We compared this cohort to the cohorts from the 2 COVID 19 time periods to identify differences caused by the COVID 19-related measures. The 3-year control period was chosen by considering the local dynamics in heart-team decision making, regularly updated guidelines and situations in which the same teams operated together during these 3 years. To assess if there was a difference in complications and outcomes in 1 of the investigated groups, we chose measures included in the department-specific quality management systems. These included the presence of a perioperative myocardial infarction as described previously [12]. Postoperative stroke was defined as an ischaemic or haemorrhagic stroke within 72 h following open-heart surgery as described previously [13]. The diagnosis was performed by the Department of Neurology. Postoperative dialysis was defined as the need for dialysis or haemofiltration following open-heart surgery in patients who did not require dialysis prior to surgery, as described previously [14]. Multiorgan failure was diagnosed by the intensive care specialists who based their decision on the Sequential Organ Failure Assessment score [15]. The presence of postoperative delirium was diagnosed by the clinical findings based on the Intensive Care Delirium Screening Checklist [16]. The diagnosis of postoperative acute respiratory distress syndrome and pneumonia was made by the intensive care specialists according to the acute respiratory distress syndrome Berlin definition [17]. In the department-specific quality management system, prolonged ventilation duration is defined as the need for intubation for more than 24 h.

### Statistical analysis

Data distribution was evaluated by plotting the standard deviation from normality, histograms, QQ plots and box plots and by performing the Shapiro-Wilks test. Data showing a parametric distribution are given as the arithmetic mean [standard deviation (SD)]. Non-parametric distributed data are given as the median [lower quartile—upper quartile]. Comparisons for parametric data were conducted using the Student *t*-test. Non-parametrically distributed data were compared using the Mann-Whitney *U* test. Categorical data were compared using the χ^2^ test. The risk adjusted odds ratios for mortality and complications were calculated using a generalized linear model adjusted for the EuroSCORE. Statistical analysis was performed using RStudio version 1.4.1103 (RStudio PBC, Boston, MA, USA) and Microsoft Excel 365 (Microsoft Corp., Redmond, WA, USA). Data comparison was facilitated with the compareGroups (Version 4.0) Package for R (R Foundation for Statistical Computing, Institute for Statistics and Mathematics, Vienna, Austria) [18].

## RESULTS

### Lockdown period

The impact of strict non-pharmaceutical interventions, also known as “lockdown”, was evaluated by comparing the lockdown period from 1 March to 15 May 2020 with the same period 3 years previously, before the COVID-19 pandemic (control period). A total of 724 patients were included; of these, 140 underwent open-heart surgery during the COVID-19 lockdown and 584 patients underwent surgery in the control period. A total of 29% fewer open-heart operations were performed during the COVID-19 lockdown.

**Figure 1:**
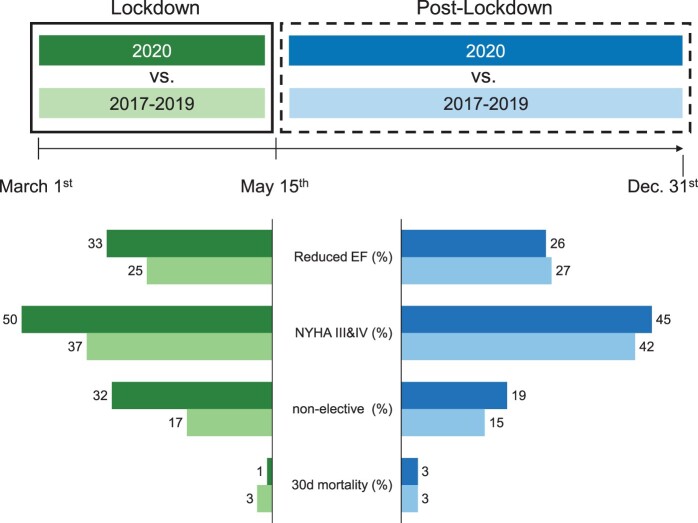
The impact of COVID-19 measures on cardiac cases at the Department of Cardiac Surgery at the Medical University of Innsbruck. Although patients who underwent surgery during lockdown were sicker and more symptomatic and the number of weekly surgical procedures significantly decreased, the implementation of a rational patient selection during resource shortages due to COVID-19 lockdown and the resulting post-lockdown period did not affect the quality of open-heart procedures and post-surgical outcomes.

The 2 groups showed no differences in presurgical medical history, comorbidities, age, sex and presence of diabetes (Table 1). Patients undergoing open-heart surgery during the COVID-19 lockdown had a significantly lower left ventricular ejection fraction, and significantly more patients were New York Heart Association functional class III and IV (Table 1) (Fig. 1). Furthermore, the number of non-elective procedures was significantly increased in patients undergoing surgery during the COVID-19 lockdown, and the median EuroSCORE was higher (Table 1) (Fig. 1).

**Table 1:** Baseline characteristics

	Lockdown period			Post-lockdown period		
	2020	2017-19	*P*-value	2020	2017-19	*P*-value
	*N = 140*	*N = 584*		*N = 600*	*N = 1693*	
Age (median IQR)	68.5 [58.0-76.0]	68.0 [60.0-76.0]	0.85	69.0 [58.0-77.0]	69.0 [59.0-76.0]	0.79
Sex (male) (%)	98 (70.0)	386 (66.1)	0.44	428 (71.3)	1119 (66.1)	0.021
BMI (median IQR)	25.0 [23.0-29.0]	26.0 [24.0-29.0]	0.33	26.0 [23.0-29.0]	26.0 [24.0-29.0]	0.04
Obese (%)	83 (59.3)	391 (67.0)	0.11	365 (60.8)	1120 (66.2)	0.022
Smoker (%)	23 (16.4)	77 (13.2)	0.39	77 (12.8)	205 (12.1)	0.70
Hypertension (%)	109 (77.9)	481 (82.4)	0.27	464 (77.3)	1321 (78.0)	0.77
PAD (%)	9 (6.43)	48 (8.22)	0.60	48 (8.00)	133 (7.86)	0.98
Diabetes (%)	38 (27.1)	151 (25.9)	0.84	144 (24.0)	427 (25.2)	0.59
Insulin treatment (%)	12 (8.57)	43 (7.36)	0.76	37 (6.17)	102 (6.02)	0.98
Dyslipidaemia (%)	102 (72.9)	439 (75.2)	0.65	435 (72.5)	1238 (73.1)	0.81
Atrial fibrillation (%)	32 (22.9)	142 (24.3)	0.80	130 (21.7)	402 (23.7)	0.33
Previous MI (%)	29 (20.7)	135 (23.1)	0.62	140 (23.3)	375 (22.2)	0.59
Prior PCI (%)	31 (22.1)	138 (23.6)	0.79	169 (28.2)	447 (26.4)	0.43
Prior cardiac surgery (%)	15 (10.7)	35 (5.99)	0.07	47 (7.85)	140 (8.27)	0.81
Creatinine clearance			0.68			0.56
> 85 ml/min	53 (37.9)	238 (40.8)		228 (38.0)	614 (36.3)	
> 50 & < 85 ml/min	60 (42.9)	253 (43.3)		260 (43.3)	757 (44.7)	
< 50 ml/min	25 (17.9)	82 (14.0)		103 (17.2)	299 (17.7)	
dialysis	2 (1.43)	11 (1.88)		8 (1.33)	23 (1.36)	
COPD (%)	12 (8.57)	58 (9.93)	0.74	47 (7.85)	125 (7.38)	0.78
Stroke (%)	7 (5.00)	32 (5.48)	0.98	39 (6.50)	105 (6.20)	0.87
PH (%)			0.45			0.33
Moderate	18 (12.9)	97 (16.6)		87 (14.5)	243 (14.4)	
Severe	10 (7.14)	32 (5.48)		23 (3.84)	91 (5.38)	
Heart failure (%)	54 (38.6)	224 (38.4)	1.00	177 (29.5)	617 (36.4)	0.003
LVEF (median IQR)	55.0 [48.0-60.0]	59.0 [50.0-63.0]	0.006	57.0 [50.0-63.0]	58.0 [50.0-64.0]	0.30
LVEF class (%)			0.22			1.00
> 50 %	94 (67.1)	436 (74.7)		442 (73.7)	1244 (73.5)	
31–50 %	39 (27.9)	125 (21.4)		127 (21.2)	362 (21.4)	
21–30 %	3 (2.14)	14 (2.40)		20 (3.33)	55 (3.25)	
≤ 20 %	4 (2.86)	9 (1.54)		11 (1.83)	32 (1.89)	
NYHA class (%)			0.021			0.023
1	29 (20.7)	129 (22.1)		143 (23.8)	337 (19.9)	
2	41 (29.3)	238 (40.8)		187 (31.2)	639 (37.7)	
3	57 (40.7)	187 (32.0)		231 (38.5)	604 (35.7)	
4	13 (9.29)	30 (5.14)		39 (6.50)	113 (6.67)	
NYHA class 3&4 (%):	70 (50.0)	217 (37.2)	0.007	270 (45.0)	717 (42.4)	0.28
EuroSCORE (median IQR)	2.35 [1.30-5.03]	2.00 [1.10-3.80]	0.011	2.20 [1.20-4.20]	2.10 [1.20-4.10]	0.64
NT-pro-BNP (median IQR) – ng/l	542 [211-1467]	526 [184-1665]	0.67	494 [164-1542]	501 [174-1772]	0.47
Troponin-T (median IQR) – ng/l	13.5 [8.8-29.1]	13.8 [8.1-25.7]	0.71	14.1 [8.5-27.8]	13.5 [8.0-24.2]	0.044
Urgency (%)			0.001			0.023
Elective	90 (68.2)	471 (82.8)		483 (80.8)	1386 (84.6)	
Urgent	31 (23.5)	65 (11.4)		81 (13.5)	165 (10.1)	
Emergency	11 (8.33)	28 (4.92)		30 (5.02)	63 (3.84)	
Salvage	0 (0.00)	5 (0.88)		4 (0.67)	25 (1.53)	
Unscheduled procedures (%)	42 (31.8)	98 (17.2)	<0.001	115 (19.2)	253 (15.4)	0.038

IQR: interquartile range; BMI: body mass index in kg/m^2^; PAD: peripheral artery disease; MI: myocardial infarction; PCI: percutaneous coronary intervention; COPD: chronic obstructive pulmonary disease; PH: pulmonary hypertension; LVEF: left ventricular ejection fraction; NYHA: New York Heart Association.

Although the number of operations performed was significantly lower, the relative numbers of the different cardiothoracic procedures, such as aortic valve replacement, transaortic valve implantation, coronary artery bypass graft (CABG), mitral valve replacement and mitral valve repair and aortic procedures did not change. The use of arterial grafts for CABG during the lockdown period was unchanged (Table 2).

**Table 2:** Surgical procedures and intraoperative details

	Lockdown period			Post-Lockdown period		
	2020	2017-2019	*P*-value	2020	2017-2019	*P*-value
	*N = 140*	*N = 584*		*N = 600*	*N = 1693*	
AVR/Avr (%)	54 (38.6)	190 (32.5)	0.21	176 (29.3)	535 (31.6)	0.33
TAVI (%)	17 (12.1)	54 (9.25)	0.38	92 (15.3)	185 (10.9)	0.006
CABG (%)	64 (45.7)	258 (44.2)	0.82	243 (40.5)	730 (43.1)	0.29
Aortic procedures (%)	22 (15.7)	76 (13.0)	0.48	76 (12.7)	202 (11.9)	0.69
ASD repair (%)	2 (1.43)	16 (2.74)	0.55	17 (2.83)	43 (2.54)	0.81
MVR/MVr (%)	21 (15.0)	114 (19.5)	0.27	128 (21.3)	348 (20.6)	0.73
TVR/TVr (%)	10 (7.14)	43 (7.36)	1.00	31 (5.17)	102 (6.02)	0.50
Transplant (%)	4 (2.86)	8 (1.37)	0.26	9 (1.50)	27 (1.59)	1.00
Arterial grafts in isolated CABG (%)			0.056			0.04
Single	28 (60.9)	113 (53.6)		127 (64.8)	322 (54.6)	
Multiple	10 (21.7)	79 (37.4)		43 (21.9)	176 (29.8)	
Perfusion time (median IQR) – min	127 [93-165]	124 [90-161]	0.63	122 [93-160]	124 [90-168]	0.28
Cross-clamp time (median IQR) – min	77.0 [60-110]	79.0 [55-105]	1.00	77.5 [55.8-102]	79.0 [54-106]	0.33

AVR: aortic valve replacement; AVr: aortic valve repair; TAVI: transcatheter aortic valve implantation; CABG: coronary artery bypass graft; ASD: atrial septal defect; MVR: mitral valve replacement; MVr: mitral valve reconstruction; TVR: tricuspid valve replacement; TVr: tricuspid valve reconstruction.

Postoperative complications did not differ from the control period except for the small but significant decrease in the total time in the ICU in patients undergoing surgery during the COVID-19 lockdown. Mortality did not differ between the lockdown period and the control period; neither did the risk adjusted mortality (Tables 3 and 4).

**Table 3:** Postoperative complications and outcomes

	Lockdown period			Post-Lockdown period		
	2020	2017-2019	*P*-value	2020	2017-2019	*P*-value
	*N = 140*	*N = 584*		*N = 600*	*N = 1693*	
Perioperative MI (%)	0 (0.00)	3 (0.51)	1.00	1 (0.17)	7 (0.41)	0.69
Postoperative stroke (%)	2 (1.43%)	7 (1.19%)	0.69	1 (0.17)	6 (0.35)	0.68
Postoperative dialysis (%)	19 (13.6)	65 (11.1)	0.51	72 (12.0)	217 (12.8)	0.66
Multiorgan failure (%)	3 (2.14)	14 (2.40)	1.00	9 (1.50)	30 (1.77)	0.80
Sepsis (%)	1 (0.71)	20 (3.42)	0.10	7 (1.17)	36 (2.13)	0.19
Postoperative delirium (%)	20 (14.3)	78 (13.4)	0.88	79 (13.2)	186 (11.0)	0.17
Postoperative ventilation duration (median IQR), h	5.0 [5.0-15.0]	6.0 [5.0-15.0]	0.42	5.0 [4.0-16.0]	6.0 [5.0-16.0]	0.003
Time in ICU (median IQR), h	20.0 [15.0-21.0]	20.0 [16.0-22.0]	0.032	20.0 [15.0-21.0]	20.0 [15.0-21.0]	0.61
Reintubation (%)	5 (3.57)	33 (5.65)	0.44	28 (4.67)	78 (4.61)	1.00
Pacemaker (%)	3 (2.14)	17 (2.91)	0.78	20 (3.33)	34 (2.01)	0.092
ARDS/Pneumonia (%)	4 (2.86)	18 (3.08)	1.00	25 (4.17)	68 (4.02)	0.97
In-hospital mortality (%)	3 (2.14)	15 (2.57)	1.00	15 (2.50)	37 (2.19)	0.78
Thirty-day mortality (%)	2 (1.43)	18 (3.08)	0.40	17 (2.83)	44 (2.60)	0.87
Readmission to ICU (%)	4 (2.86)	18 (3.08)	1.00	22 (3.67)	38 (2.24)	0.08
Rehospitalization (%)	16 (11.4)	53 (9.08)	0.49	42 (7.00)	107 (6.32)	0.63
Prolonged ventilation duration (%)	25 (17.9)	84 (14.4)	0.37	93 (15.5)	263 (15.5)	1.00

IQR: interquartile range; MI: myocardial infarction; ICU: intensive care unit; ARDS: acute respiratory distress syndrome.

**Table 4:** Odds ratio for mortality and complications in the lockdown and post-lockdown periods adjusted by EuroSCORE

	Lockdown period		Post-Lockdown period	
	Odds ratio		Odds ratio	
	OR (95% CI)	*P*-value	OR (95% CI)	*P*-value
Mortality	0.39 (CI : 0.06-1.42)	0.22	1.04 (CI : 0.57-1.81)	0.90
Perioperative MI	^*^		0.36 (CI : 0.02-2.09)	0.35
Postoperative stroke	1.07 (CI : 0.26-7.27)	0.93	2.45 (CI : 0.40-47.71)	0.42
Postoperative dialysis	1.07 (CI : 0.58-1.90)	0.82	0.86 (CI : 0.62-1.16)	0.33
Multiorgan failure	0.84 (CI : 0.19-2.64)	0.79	0.75 (CI : 0.33-1.56)	0.47
Sepsis	0.19 (CI : 0.01-0.92)	0.11	0.45 (CI : 0.17-0.99)	0.07
Postoperative delirium	1.00 (CI : 0.57-1.69)	1.00	1.20 (CI : 0.89-1.58)	0.22
Reintubation	0.56 (CI : 0.18-1.36)	0.24	0.95 (CI : 0.59-1.47)	0.82
Pacemaker	0.72 (CI : 0.17-2.18)	0.60	1.62 (CI : 0.90-2.81)	0.10
ARDS/pneumonia	0.90 (CI : 0.26-2.47)	0.85	1.01 (CI : 0.62-1.60)	1.0
Readmission to ICU	0.89 (CI : 0.25-2.44)	0.83	1.62 (CI : 0.93-2.74)	0.08
Rehospitalization	1.26 (CI : 0.68-2.23)	0.45	1.10 (CI : 0.75-1.58)	0.62
Prolonged ventilation duration	1.12 (CI : 0.65-1.87)	0.68	0.95 (CI : 0.72-1.24)	0.70

* There was no perioperative MI in this time period.

OR: odds ratio; 95% CI: 95% confidence interval; MI: myocardial infarction; ICU: intensive care unit; ARDS: acute respiratory distress syndrome.

The median waiting time for open-heart surgery in the lockdown period was significantly longer than in the control period. The patients waited a median of 65.5 [32.8–93.8] days in the lockdown period and 29.5 [16.0–55.8] days in the control period (<0.001).

### Post-lockdown period

The post-lockdown period included all open-heart procedures performed between 16 May and 31 December 2020. Data were compared with the data from the same period 3 years previously (control period).

Patients undergoing open-heart surgery during the post-lockdown period were more often male and had a lower body mass index. Other patient demographics, e.g. diabetes, smoking and hypertension, were the same in both groups (Table 1).

Significantly fewer patients undergoing open-heart surgery in the post-lockdown period had heart failure, but the number of symptomatic patients (New York Heart Association functional class III and IV) did not differ between the post-lockdown and the control period (Table 1) (Fig. 1). The number of aortic valve replacement, CABG, mitral valve replacement/mitral valve repair and aortic procedures did not change whereas the transaortic valve implantation procedures increased. There was a significant reduction in the use of arterial grafts for CABG in the post-lockdown period (Table 2).

There was no difference in post-procedural complications and outcomes except the postoperative ventilation duration was lower in patients undergoing open-heart surgery in the post-lockdown period. Mortality did not differ between the post-lockdown period and the control period; neither did the risk adjusted mortality (Tables 3 and 4).

The median waiting time for open-heart surgery in the post-lockdown period was significantly longer than in the control period. Patients waited a median of 51.0 [19.0–87.0] days in the post-lockdown period and 28.0 [15.0–47.0] days in the control period (<0.001).

## DISCUSSION

In addition to non-pharmaceutical interventions and vaccinations, combating the severe acute respiratory syndrome coronavirus 2 pandemic relies on shifting medical resources towards treatment of patients with COVID-19. Inter alia, this comes at the expense of cardiac surgery caseloads; hence, the so-called collateral damage of non-COVID cardiac (and other elective) surgery patients is much discussed. The medical community lacks knowledge about the extent of this collateral damage as well as experience in facing a shortage of resources that are otherwise taken for granted [1]. We have quantified the effect of COVID-19 on cardiac surgery programmes by comparing the lockdown and the subsequent post-lockdown periods with the same period in previous years at our institution, the goal being to learn from the current situation for future periods of resource shortages of any nature. The lockdown period was defined as the 15 weeks between 1 March and 15 May 2020. Although the wait list for cardiac surgical procedures increased because of a drastic reduction of cases performed during the lockdown period, outcomes and perioperative data remained stable not only during that period, but also during the following months. These observations indicate that the measures implemented in our institution did not worsen the outcome of patients undergoing open-heart surgery compared to the control cohorts because neither the perioperative morbidity nor mortality changed. Multiple factors including but not limited to operating room capacity, ICU capacity, human resources and equipment led to a reduction in cardiac cases. At our institution, primarily a compromise had to be made in relation to ICU capacity allocation between COVID-19 patients, urgent cardiac surgery patients and patients waiting for elective cardiac surgery. In order to deal with the influx of patients with COVID-19, elective cardiac surgery cases were reduced. Numerous actions were taken to compromise with ICU capacity for patients with COVID-19, patients having urgent cardiac surgery and patients waiting for elective cardiac surgery. These measures included amongst others: (i) temporary suspension of teaching cases, (ii) minor progress of elective cases, (iii) effort to minimize ICU and (iv) overall hospital stay whenever possible. Each allocated patient was triaged by an experienced consultant. Highly symptomatic patients were prioritized. Minor ICU capacities were reserved for patients with serious risks for a prolonged postoperative course. Many guidance statements for cardiac surgeons suggested similar interventions [3, 4, 9, 19, 20]. These actions seemed to be sufficient regarding the perioperative and short-term outcomes: Although patients who underwent cardiac surgery during the lockdown period were much more symptomatic, had a lower left ventricular ejection fraction and a higher EuroSCORE, the cross-clamp time, ICU stay and 30-day mortality did not differ compared to the same period in previous years (Fig. 1).

Although both caseload and case-mix reached prelockdown levels within a few weeks after restrictions were lifted and COVID-19 cases declined (“end of first wave”), the lasting effect of the lengthened wait list remained unclear. We therefore compared the post-lockdown period, defined as the time between 16 May 2020 and 31 December 2020, with the same period in previous years. Except for a shorter postoperative ventilation time in the 2020 cohort, periprocedural data did not differ. Again, the results indicated that the anticipated periprocedural collateral damage could be avoided.

This apparent discrepancy between our findings and other widely hypothesized collateral damage effects [21, 22] is potentially based on the major limitation of this study.

Meanwhile, according to emerging reports [23], an increasing number of patients avoided medical care while being symptomatic and deteriorating and even dying at home. Overall, it will be extremely difficult to quantify the pandemic’s impact on COVID-19 and non-COVID-19 morbidity and mortality because these data fall into 3 different main categories. The first category is the number of infected patients and their clinical course, which is objectively quantifiable and almost globally available to date [24]. The second category is the most difficult to obtain, namely, the number of non-COVID-19 patients avoiding medical care. Underlying reasons might be patient related, e.g. misinterpretation of symptoms such as chest discomfort or dyspnoea, while system-related factors like instructions to stay at home or fear of infection in a medical facility contributed additionally [21].

The maximum potential to close the gap in evidence lies in the third category of data, i.e. the number of non-COVID-19 patients undergoing medical interventions since the outbreak of the pandemic. Reports on these patients, as in our study, are sparse. Still a lot can be learned from the COVID-19 situation: e.g. a Swedish group discovered that in their cohort the number of myocardial infarctions indeed decreased as well as the number of patients seeking treatment for myocardial infarction [25]. Amongst others, policy makers, academics and healthcare providers should be urged to publish their insights to adapt and make more specific guidelines for the future.

The limitation of this study is that only data about patients who underwent surgery are currently available. The actual unreported mortality is difficult to measure. In a personal communication with the responsible administrative staff member, we identified 6 patients who died while waiting for surgery during the lockdown period. However, the numbers must be interpreted with caution. We were only able to identify patients who died in one of our accompanied Tyrolean hospitals. Patients who died at home or in a non-accompanied hospital are missing. Another limitation of this trial is that patients undergoing alternative procedures for treating coronary artery disease, interinstitutional transfers and out-of-hospital survival were not evaluable. These limitations might also have affected the analysis of the wait list.

In summary, we have shown that the implementation of the above-mentioned measures did not lead to impaired outcomes after cardiac surgery during lockdown at our institution. Future pandemic situations might be managed accordingly. Cardiac surgery teams should be encouraged to critically revise both their own and otherwise reported COVID-19 related selection measures to brace for future periods of limited healthcare resources.

### Funding statement

None.

**Conflict of interest:** Authors have no conflict of interest.

## Authors’ contributions

**Felix Nägele:** Conceptualization; Data Curation; Formal Analysis; Methodology; Investigation; Validation; Writing—original draft; Writing—review & editing. **Clemens Engler:** Conceptualization; Data Curation; Formal Analysis; Methodology; Investigation; Validation; Writing—original draft; Writing—review & editing. **Michael Graber:** Conceptualization; Data Curation; Writing—review & editing. **Nina Remmel:** Formal analysis; Data Curation; Investigation; Writing—review & editing. **Jakob Hirsch:** Data Curation; Writing—review & editing. **Leo Pölzl:** Data Curation; Writing—review & editing. **Rosalie Huber:** Data Curation; Writing—review & editing. **Victor Schweiger:** Data Curation; Writing—review & editing. **Juliane Kilo:** Data Curation. **Ivan Tancevski:** Writing—original draft; Writing—review & editing. **Michael Grimm:** Writing—review & editing. **Can Gollmann-Tepeköylü:** Writing—original draft; Writing—review & editing. **Johannes Holfeld:** Project administration; Supervision; Writing—original draft; Writing—review & editing.

## Data availability statement

The data underlying this article will be shared on reasonable request to the corresponding author.

## Abbreviations

CABG: coronary artery bypass graft
COVID-19: coronavirus disease 2019
ICU: intensive care unit
TAVI: transaortic valve implantation
